# Oligosecretory Myeloma With Amyloidosis and Alopecia

**DOI:** 10.1177/2324709617752737

**Published:** 2018-01-24

**Authors:** Anum Bilal, Paul Der Mesropian, Franklin Lam, Gulvahid Shaikh

**Affiliations:** 1 Albany Medical Center, Albany, NY, USA; 2 Albany Stratton VA Medical Center, Albany, NY, USA

**Keywords:** amyloidosis, alopecia universalis, nonsecretory multiple myeloma, multiple myeloma

## Abstract

Amyloidosis is a systemic illness characterized by the extracellular deposition of abnormal proteins in body tissues and organs. In addition to renal involvement, amyloidosis can also present with a variety of skin manifestations, though rarely with alopecia. Sixteen cases of alopecia secondary to systemic amyloidosis are reported. There is one reported case that presented with alopecia universalis. We report a case of a 68-year-old woman presenting with alopecia universalis, rapid decline in kidney function, and nephrotic syndrome who was found to have multiple myeloma-associated AL amyloidosis (immunoglobulin light chain). Her serological workup including serum electrophoresis was negative and she underwent renal biopsy. Pathology revealed eosinophilic material within the mesangium that was Congo-red positive, had apple-green birefringence under polarized light, and ultramicroscopically appeared as fibrillary material. Subsequent bone marrow examination showed a diffuse increase in plasma cells with atypia indicating plasma cell neoplasm. This case underlines several interesting aspects of multiple myeloma and the way it may present with amyloidosis. The lack of monoclonal spike on electrophoresis yet positive light chain analysis deserves special attention by clinicians to avoid a missed diagnosis. The extensive skin involvement also raises several questions regarding the pathologic mechanisms of alopecia in a patient with amyloidosis.

## Introduction

Amyloidosis results from the deposition of fibrils composed of low-molecular-weight subunits of a variety of proteins in the extracellular matrix. Amyloid deposits, regardless of clinical pathologic variant or the tissue involved, are composed of 7.5 to 10 nm rigid, linear, nonbranching, aggregated, paired fibrils and classically have an “apple green” appearance when stained with Congo-red under polarized light.^1,2^ The modern classification of this disease is based on the nature of the precursor protein.^3^ At least 30 such proteins have been reported.^4,5^

AL amyloidosis (immunoglobulin light chain) is an uncommon disorder and the exact incidence is unknown. In the United States, the incidence appears to be stable at approximately 6 to 10 cases per million person-years.^6^ The median age at diagnosis is 64 years and less than 5% of patients are younger than 40 years. A higher proportion of men are affected, accounting for 65% to 70% of patients.^6^ AL amyloidosis may result from the overproduction of immunoglobulin light chains, which can occur in monoclonal gammopathies, such as multiple myeloma (MM), as well as certain lymphoproliferative diseases.^7^ In the kidney, amyloid most commonly deposits in the glomeruli,^2^ leading to significant proteinuria and loss of glomerular filtration rate; less commonly, crescentic glomerulonephritis,^8^ vascular and tubular deposition,^9^ and cast nephropathy^10^ may occur.

Systemic amyloidosis can manifest with a variety of skin conditions. Signs of skin involvement in systemic amyloidosis include waxy thickening, easy bruising (ecchymoses), and subcutaneous nodules or plaques. Purpura characteristically elicited in a periorbital distribution (raccoon eyes) by a Valsalva maneuver or minor trauma is present in only a minority of patients but is highly characteristic of AL amyloidosis. Infiltrative lesions, blisters, paronychia, and Sjogren-like syndrome have also been reported.^4^ The incidence of clinical skin involvement is estimated at 29% to 40% of all cases of systemic amyloidosis.^11,12^

## Case Report

**Figure 1.**
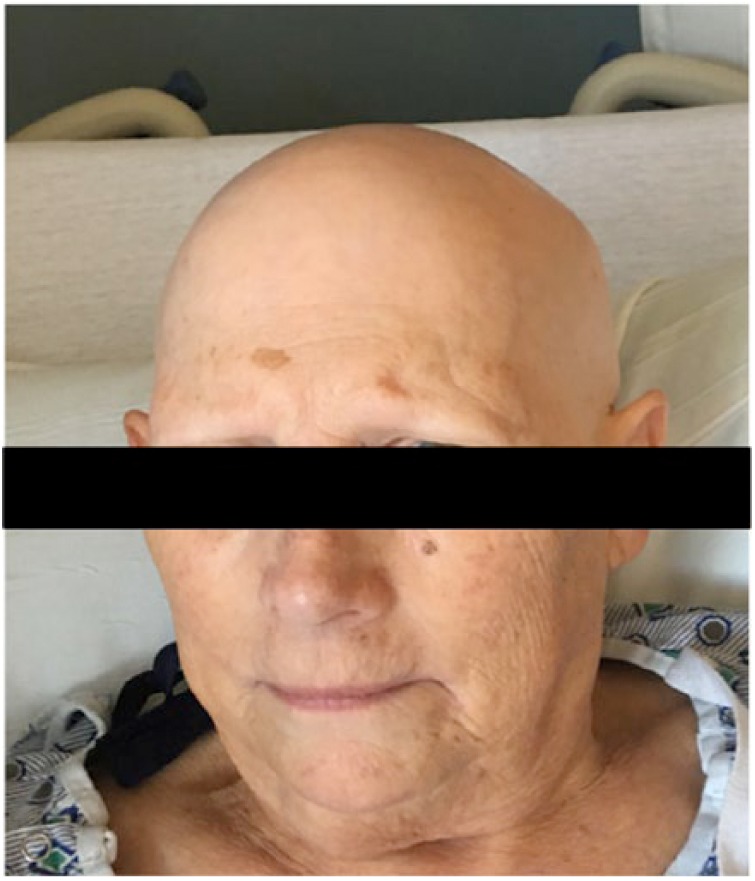
Scalp picture showing alopecia.

**Figure 2.**
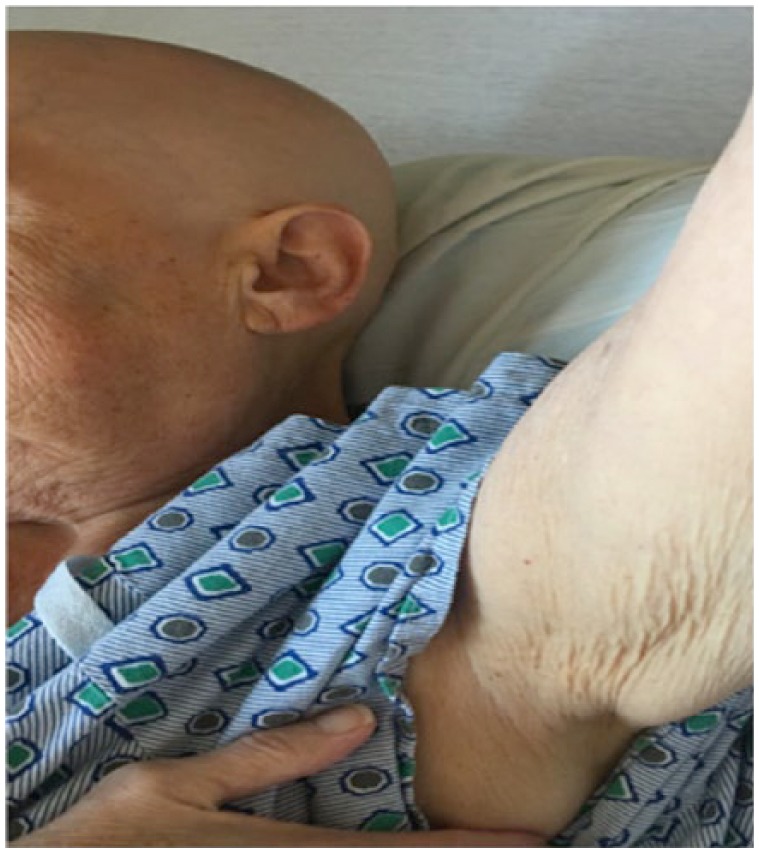
Picture showing hair loss of arm, underarm.

We present a case of a 68-year-old white female who presented with the complaint of fatigue and hair loss for several months. She had a past medical history of benign essential hypertension, bradycardia s/p (status post) cardiac pacemaker secondary to Mobitz type 2 atrioventricular block, hyperlipidemia, essential tremor, eczema, and osteopenia. An evaluation by dermatology had confirmed a diagnosis of alopecia totalis. This progressed to complete hair loss over the next few months. She was referred to nephrology for worsening creatinine (0.9-1.9 mg/dL over the course of 1 year) and proteinuria. On physical examination, she exhibited normal vital signs. Skin examination revealed complete hair loss of the body including the scalp (Figure 1), eyebrows, eyelashes, and axillary (Figure 2) and pubic hair. Cardiac and lung examinations were unremarkable although she was noted to have trace of lower extremity edema.

**Figure 3.**
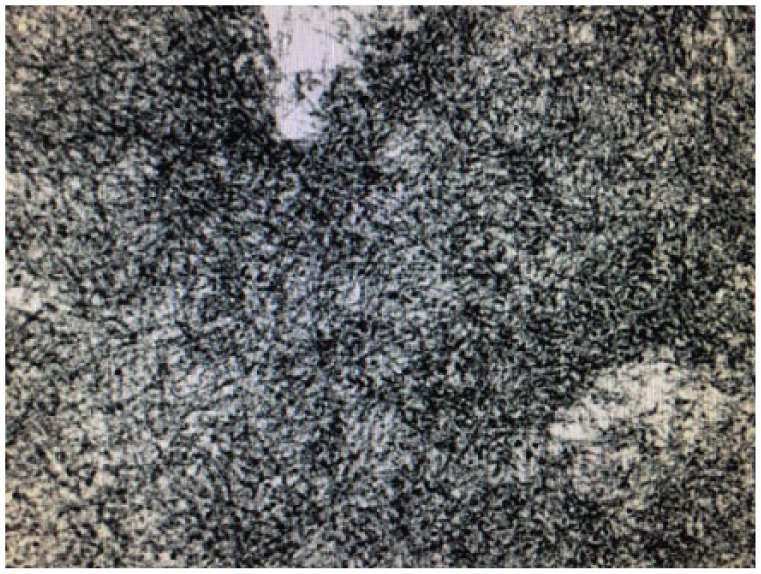
Electron microscopy picture showing fibrils.

Urinalysis disclosed significant albuminuria, albumin/creatinine ratio of 8 g/g of creatinine, and urine protein/creatinine ratio of 9.8. Serum albumin was low at 3 g/dL. Urine microscopic examination was bland. Serological workup including anti-Ro, anti-La, anti-RNP, anti-Sm, anti Scl-70, anti Jo-1, ANA, anti–double stranded DNA, ANCA screen, C3, C4, hepatitis profile, HIV screen, cryoglobulins, and rheumatoid factor were all negative. Serum and urine protein electrophoresis revealed no paraproteins. She was noted to be anemic with hemoglobin of 12 g/dL. Given declining renal function, significant proteinuria, and a negative workup, renal biopsy was pursued. This revealed eosinophilic material in the mesangium with apple-green birefringence on Congo-red stain. Deposition of fibrillary material was seen in electron microscopy (Figure 3). Immunohistochemistry of the biopsy specimen confirmed the presence of amyloid, AL type (kappa). Serum-free light chain analysis demonstrated an elevated kappa/lambda ratio of 79.8; kappa concentration was 1014 mg/dL, while free lambda was 12.7 mg/dL. Bone marrow examination showed 28% plasma cells with atypia 46, XX; fluorescence in situ hybridization demonstrated monosomy-13 with gains in chromosomes 9 and 11.

The patient was initiated on bortezomib and dexamethasone treatment for amyloidosis associated with MM. She was hospitalized 1 month later for symptoms of heart failure and was found to have a reduced left ventricular ejection fraction of 25%. Cardiac dysfunction was presumed to be secondary to amyloidosis. Her renal failure continued to progress and required initiation of hemodialysis. She was switched to ixazomib because of severe adverse effects to bortezomib after 3 cycles. She had a very good partial response to chemotherapy with serum kappa light chain level decreasing from 1854 mg/dL to 165 mg/dL. Renal function failed to recover and she remained dialysis dependent. She experienced an improvement in alopecia with new hair growth following the initiation of chemotherapy coincident with a significant reduction in the kappa light chain levels. The favorable response to chemotherapy may be predictive of an improved overall prognosis in our patient.

## Discussion

The incidence of alopecia as a manifestation of systemic amyloidosis is unknown. However, this may be because of underreporting as it is not a well-recognized manifestation of amyloidosis. On review of the literature, we discovered 16 cases of amyloidosis with associated hair loss on examination.^13-19^ There is one reported case of alopecia universalis as the initial presentation of an occult amyloidosis. Wheeler et al^14^ described a case of a woman with anonychia, generalized hair loss, and weight loss for 18 months who was hospitalized with symptoms of heart failure and found to have amyloidosis associated with MM. Among the case reports of alopecia secondary to amyloidosis, only 2 cases reported by Hunt et al^16^ and Bedlow et al^17^ described renal failure developing after diagnosis of amyloidosis. In this article, we report a similar case that is remarkable in presentation with alopecia universalis as the earliest indicator of amyloidosis and subsequent development of renal failure.

Multiple myeloma is characterized by proliferation of plasma cells and production of monoclonal proteins. In certain cases, light chain overproduction is detected where monoclonal protein production is not, termed nonsecretory multiple myeloma (NSMM) or oligosecretory multiple myeloma. This phenomenon was first described by Serre in 1958 and estimated incidence is 1% to 5%.^20^ Ten percent of all newly diagnosed cases in a large cohort at Mayo Clinic including more than 1000 patients was found to have oligosecretory MM.^21^ Kappa variant is 4 times more common than lambda involvement in individuals with NSMM.^22^ Our patient had kappa chain predominance.

Gafumbegete et al^23^ described 2 variations of NSMM: the first is one that “produces” immunoglobulins without secreting them out of the cell, and the second is a “nonproducer” variant. In this case the plasma cells lose the ability to form immunoglobulins. In 1976, Preud’Homme et al^24^ hypothesized that a chain defect predisposing to expeditious enzymatic breakdown of the immunoglobulin results in the serum or urinary immunoglobulin becoming indiscernible. Other causes of nonproduction of immunoglobulins in MM may be loss of immunoglobulin synthesis,^25^ lack of normal intracellular immunoglobulin transport and processing or release of antigenically distorted products.^26^

Osserman and Takatsuki^27^ suggested that a decline in serum γ-globulin concentration found in each case of NSMM as the only difference between MM and NSMM. In another case series, almost all individuals had depleted plasma levels of all immunoglobulin classes, and all had low plasma levels of at least one immunoglobulin class.^28^ Our case had low levels of IgM while IgG and IgA levels were within normal limits.

MM occurs in association with amyloidosis in 10% to 15% of cases.^29^ Additionally, the monoclonal light chain type in MM presenting with amyloidosis is of the lambda type in approximately 75% of cases, the remaining are kappa type.^30^ In the case we presented, kappa chains were elevated as opposed to lambda chains, the more common finding in myeloma-associated amyloidosis.

When MM and AL amyloidosis are diagnosed in the same patient, the myeloma is typically diagnosed before or around the time of the diagnosis of AL amyloidosis.^31^ Less commonly, myeloma develops more than 6 months after the diagnosis of amyloid. This is termed as delayed progression. The Mayo Clinic reported a series of 1596 patients with AL amyloidosis presenting between 1960 and 1994. They found only 6 patients (0.4%) showed delayed progression (at 10 to 81 months) to overt myeloma.^32^ This usually occurred in patients lacking cardiac or hepatic amyloid who lived long enough to develop myeloma. In another series of 4319 patients seen at the Mayo Clinic between 1990 and 2008 with a diagnosis of myeloma who had at least 6 months of follow-up, there were 47 patients (1.1%) in whom the diagnosis of AL amyloidosis followed the diagnosis of myeloma by at least 6 months.^32^ The outcomes of these patients were poor, especially in those with cardiac involvement, with a median survival after the diagnosis of AL amyloidosis of 9 months (95% confidence interval = 4-14 months).

Our patient had severe and progressive renal failure necessitating hemodialysis. Her large amount of proteinuria at baseline in renal AL amyloidosis represents a poor prognostic factor for renal response. However, she had favorable light chain response to therapy, which should bode well for renal prognosis. In a series of 122 patients with AL amyloidosis with renal involvement, it was shown that hematologic response (defined as a 50% reduction in serum monoclonal protein or free light chain) was highly correlated with renal response—hematologic response was present in 96% of renal responders versus only 54% of renal nonresponders.^33^

Our patient additionally had a presentation of severe alopecia, which is considered classically to be an autoimmune process. There was no evidence of an alternate immune process to explain this alopecia. The etiology of alopecia in amyloidosis was postulated by Hunt et al^16^ to be secondary to vascular impairment with inhibition of anagen restoration based on absence of inflammation on skin biopsy. Miteva et al^13^ reported trichoscopic findings in alopecia associated with systemic amyloidosis. The authors postulated the concept that mechanical constriction of the follicles caused by abnormal follicular or perifollicular deposition of amyloid may lead eventually to anagen arrest. The deposition of amyloid throughout the skin to cause extensive hair loss to the degree seen in our patient has not been previously reported in the literature. The response to chemotherapy in our case suggests that the alopecia was, in fact, related to amyloidosis interfering with the hair growth process.

A dual diagnosis of NSMM with an AL amyloidosis has been reported as individual cases but literature is scarce. The amyloid fibrils deposited in AL amyloidosis are made up of monoclonal immunoglobulin light chains secreted by the responsible plasma cell clone. It has been recognized by Azar et al^34^ that a well-honed endoplasmic reticulum is a feature of cells that are able to produce large quantities of protein intended for transfer. The recognition of amyloid deposits either intracellularly or extracellularly helped form the hypothesis that plasma cells may also be capable of excreting immunoglobulins, which then gather to form amyloid fibrils.^23^

The most interesting finding, in this case, was the negative report for serum paraproteins and absence of M-protein spike on urine electrophoresis. M-spike is present in only 82% of cases without immunofixation.^35^ This test is less sensitive than the nephelometric testing for free light chains, which is essential for diagnosis.^36^ When MM is suspected, free light chain testing should be done along with immunofixation. Even with additional testing, 3% of cases are reported as “nonsecretory.”^22^ This case highlights the importance of testing for free light chains, as testing for serum and urine protein electrophoresis alone can miss the diagnosis of an oligosecretory myeloma.
